# Warming Ocean Conditions Relate to Increased Trophic Requirements of Threatened and Endangered Salmon

**DOI:** 10.1371/journal.pone.0144066

**Published:** 2015-12-16

**Authors:** Elizabeth A. Daly, Richard D. Brodeur

**Affiliations:** 1 Cooperative Institution for Marine Resources Studies, Oregon State University, Newport, Oregon, United States of America; 2 Fish Ecology Division, Northwest Fisheries Science Center, National Marine Fisheries Service, National Oceanic and Atmospheric Administration, Newport, Oregon, United states of America; The Evergreen State College, UNITED STATES

## Abstract

The trophic habits, size and condition of yearling Chinook salmon (*Oncorhynchus tshawytscha*) caught early in their marine residence were examined during 19 survey years (1981–1985; 1998–2011). Juvenile salmon consumed distinct highly piscivorous diets in cold and warm ocean regimes with major differences between ocean regimes driven by changes in consumption of juvenile rockfishes, followed by several other fish prey, adult euphausiids and decapod larvae. Notable, Chinook salmon consumed 30% more food in the warm versus cold ocean regime in both May and June. Additionally, there were about 30% fewer empty stomachs in the warm ocean regime in May, and 10% fewer in warm June periods. The total prey energy density consumed during the warmer ocean regime was also significantly higher than in cold. Chinook salmon had lower condition factor and were smaller in fork length during the warm ocean regime, and were longer and heavier for their size during the cold ocean regime. The significant increase in foraging during the warm ocean regime occurred concurrently with lower available prey biomass. Adult return rates of juvenile Chinook salmon that entered the ocean during a warm ocean regime were lower. Notably, our long term data set contradicts the long held assertion that juvenile salmon eat less in a warm ocean regime when low growth and survival is observed, and when available prey are reduced. Comparing diet changes between decades under variable ocean conditions may assist us in understanding the effects of projected warming ocean regimes on juvenile Chinook salmon and their survival in the ocean environment. Bioenergetically, the salmon appear to require more food resources during warm ocean regimes.

## Introduction

Understanding how climate change will affect the relationship between the ocean environment and salmon survival is important for the conservation of threatened and endangered Pacific salmon. Chinook salmon (*Oncorhynchus tshawytscha*) in the Northern California Current (NCC) on the west coast of the United States have numerous Distinct Population Segments (DPS) currently federally listed as threatened or endangered. Salmon move from their freshwater rearing habitat to the marine environment as juveniles, where ocean conditions that are encountered can determine their ultimate success and return as adult salmon to their natal rivers [1]. The cyclical interannual and interdecadal ocean-atmospheric variability of the NCC has been closely coupled to Pacific salmon survival [2–4]. Climate change, whereby average environmental conditions are increasing or decreasing for an extended period of time superimposed upon normal ocean variability, can create intense shifts in survival, distribution, and biomass of organisms in marine ecosystems [5–8]. By understanding the mechanisms of fluctuating marine survival, especially under the constraints of increasing ocean temperatures, fishery managers can improve the effectiveness of freshwater mitigation and hatchery release practices.

Extensive analyses on the connection between salmon recruitment and marine environmental conditions have been conducted in the Northeast Pacific Ocean [4,9–12]. In the NCC, salmon recruitment has been shown to have a negative or dome-shaped relationship with ocean temperature [13–15], whereas salmon stocks from northern regions of the Northeast Pacific generally exhibit positive correlations [15,16]. The mechanisms behind increased ocean temperatures and decreased survival of juvenile Chinook salmon appear to be closely aligned with bottom up processes of food availability [4,17], although there is generally a paucity of top-down studies to date in the nearshore ocean [4,18]. Long term data sets of juvenile and adult salmon marine feeding have shown interdecadal changes in diet composition and stomach fullness [19–23], but linking these changes to ocean temperature changes is critical to understanding how global climate change may impact salmon populations.

In ecotherms, basal metabolic rates increase exponentially with increases in temperature, and ectotherms can actually grow faster in warmer water if prey biomass is sufficient for the increased caloric demands associated with higher metabolism costs. Studies using bioenergetics models have shown that food consumption and growth of salmon can be affected by environmental temperature, fish size and stomach evacuation rate, ration size, prey type and quality [21, 24–26]. Bioenergetic modelling of growth and temperature suggests an optimal temperature for growth, but one that declines as daily rations decline [27]. Gut evacuation rates also increase with increasing water temperature [28,29], thus requiring more food resources in a warmer ocean.

We utilize a long-term study (19 study years spanning three decades) of salmon feeding and condition to investigate three possible mechanisms to explain lower survival of Chinook salmon during warmer ocean regimes by examining changes in: 1) diet composition and amount of food eaten, 2) energy density consumption, and 3) measures of fish health and fitness using length and condition factor of the fish at capture. Trophic and physical characteristics were evaluated along with regional climate indices, and we expect that during warmer ocean regimes, juvenile salmon will consume a different prey community, less total amount of food, lower energetic density prey, and will exhibit lower condition and lower adult returns from the ocean.

## Materials and Methods

### Juvenile salmon collection methods

**Fig 1:**
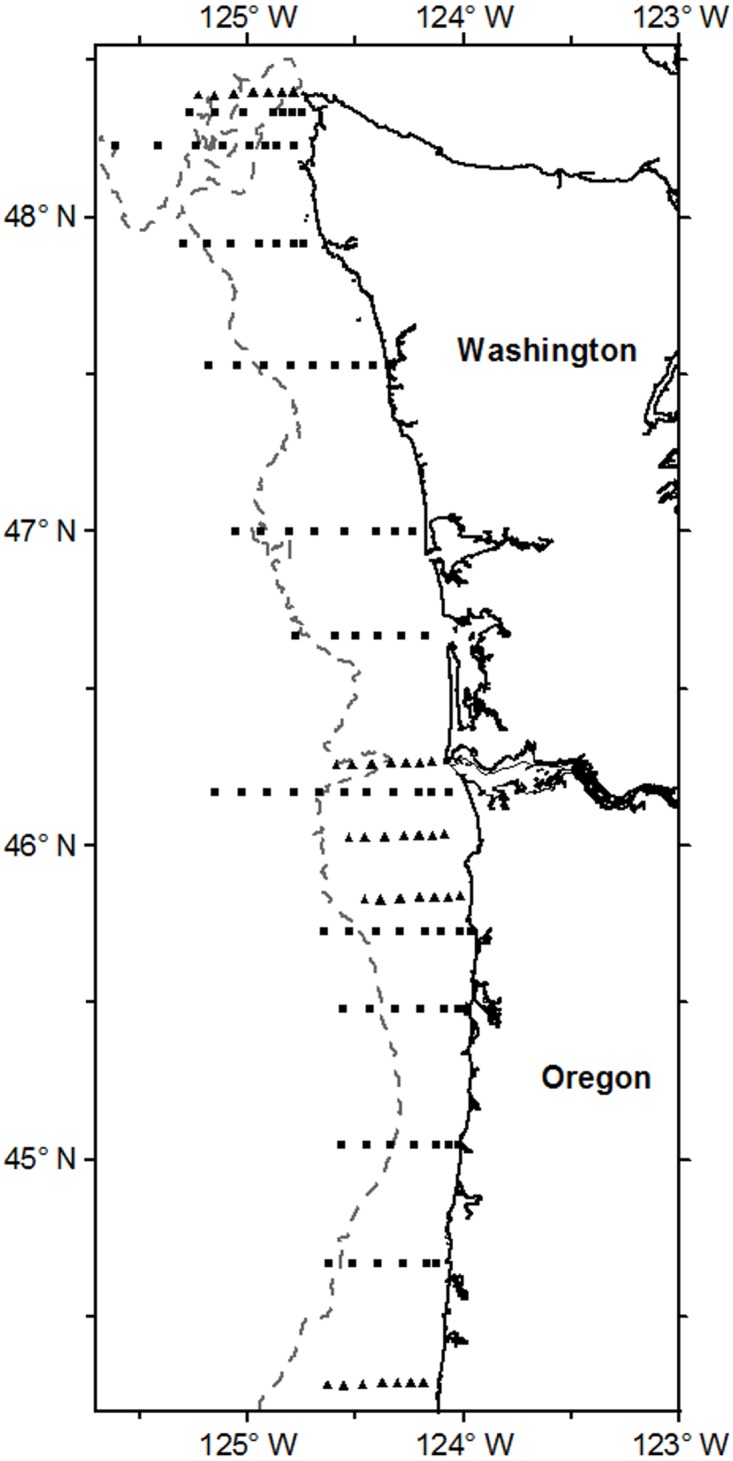
Ocean sampling stations along the Oregon and Washington coast for juvenile Chinook salmon. Map with general station locations off the coast of Oregon and Washington in the USA where juvenile salmon were sampled in 1981–1985 from OSU study as triangles and 1998–2011 during the Plume Study as squares. North-South dashed line reflects the 200 m isobaths line. Map created using **Surfer** ^®^ - Golden Software, LLC.

Juvenile salmon were collected from two different sampling programs: a program from 1981–1985 conducted by Oregon State University (OSU study) which used fine-mesh purse seines to collect juvenile salmon in coastal waters [30,31], and a long term study by the Estuarine and Ocean Ecology group of the National Oceanic and Atmospheric Administration (NOAA) Fisheries (1998–2011; Plume Study) which used surface trawling to collect juvenile salmon in the Columbia River Plume and coastal waters (Table 1). Sampling stations for both studies were generally located along predetermined transect lines running perpendicular to shore (Fig 1). Not all years or months were surveyed in their entirety (Table 1). For the OSU study, fine mesh (32-mm) herring purse-seines were fished along east-west transects from Cape Flattery off northern Washington to Cape Blanco off Oregon. The net size varied from 457 m to 495 m in length and 20 m to 60 m in depth. Most of these collections were conducted during daylight. For the Plume study, juvenile salmon were collected using a Nordic 264 pelagic rope trawl, which has a mouth opening 30 m wide by 20 m deep, fitted with a 0.8-cm cod-end liner. The rope trawl was towed at the surface during daylight for 30 minutes at approximately 6 km/h. All captured salmon were identified to species, measured (fork length to the nearest 1 mm), checked for adipose fin clips (Plume study only), individually labeled, and immediately frozen. In the laboratory, field identifications of salmon were verified, and each salmon was remeasured and weighed. Stomachs were removed and placed in a 10% formaldehyde solution or a non-formaldehyde preservative for approximately 2 weeks, then rinsed with fresh water for 24 h before being transferred into 70% ethanol. All animal work was conducted according to relevant national guidelines. Fish were collected under the Endangered Species Act (ESA) Section 10 permit #1410–7A, which is the federal procedure for research directed by NOAA that includes ESA-listed species. Neither NOAA nor OSU collections of fishes required a separate review by an Institutional Animal Care and Use Committee.

**Table 1:** Study period with regime condition, study dates, and sample size of yearling Chinook salmon collected in May and June.

Study & Year	Regime	May Cruise dates	Yearling Chinook Salmon N =	June Cruise dates	Yearling Chinook Salmon N =
OSU					
1981	Warm	16–25	55	9–18	18
1982	Cold	19–24, 27–2	97	7–22	220
1983	Warm	16–27	88	9–16, 22–27	24
1984	Warm	-	-	4–20	78
1985	Warm	29–31	134	1–5, 10–25	176
Plume					
1998	Warm	-	-	16–25	30
1999	Cold	18–25	308	16–24	190
2000	Cold	22–24	134	17–25	93
2001	Cold	20–22	74	24- July 1	40
2002	Cold	21–22, 28	186	21–28	131
2003	Warm	20–23	109	24- July 3	131
2004	Warm	22–25	97	22–29	85
2005	Warm	*	*	12–22	21
2006	Warm	24–30	255	19–28	91
2007	Cold	24–30	251	21–28	132
2008	Cold	23–29	629	22–29	467
2009	Cold	23–29	481	23–30	166
2010	Warm	21–27	261	21–28	160
2011	Cold	21–27	219	20–27	98

Sampling year, ocean regime, and cruise sampling dates for May and June in which yearling Chinook salmon were captured along with their sampled number. OSU refers to a program conducted by Oregon State University and Plume refers to the study by the Estuarine and Ocean Ecology group of NOAA Fisheries.

*Low sample size.

### Climatic indices and adult salmon returns as an index of survival

Previous publications on juvenile salmon in our sampling region have identified significant May and June differences in biological metrics of the juvenile salmon; therefore, we analyzed separately the fish metrics by month for our study [20,32]. In addition, as Chinook salmon enter the ocean and rapidly migrate north [33], we do not expect that the fish caught in May are the same individuals as the fish caught in June, giving further justification to treating each sampling month separately in our analysis.

Juvenile salmon trophic (diet composition, stomach fullness, and salmon energetic intake per meal), and condition (length and length-weight condition factor) metrics were compared to commonly used indices of NCC climatic variability (S1 Table) including: Sea Surface Temperature (SST), Pacific Decadal Oscillation (PDO), North Pacific Gyre Oscillation (NPGO), upwelling index (UPW), Eastward Ekman transport (EET), and Columbia River flow (COR). Several measures of local and regional ocean temperature were utilized including SST, PDO, which is an index based on the long term variation in sea surface temperature [2] with more positive values indexing warmer ocean conditions, and NPGO which is an index correlated with fluctuations in salinity, nutrients and chlorophyll-*a*. The NPGO is the second Empirical Orthogonal Function of the anomalies of the monthly mean SST in the North Pacific Ocean, (PDO being the first; [2]). Positive NPGO values of relate to more productive colder ocean conditions. The UPW index is a measure of coastal wind speed on the surface which causes the surface layer to offshore, being replaced with deeper, colder, and nutrient rich upwelled water. The EET index is an index of water circulation and transport. Lastly, COR is a measure of fresh water input into the Northern California Current from the Columbia River and is related to the subsequent ocean plume development [34]. Annual physical metrics were averaged for the spring months of March, April and May to compare with May biological data and April, May and June to compare with June biological data. Using the climatic conditions just prior to and during out migration of juvenile salmon was viewed as a proxy of the ocean regime that the salmon first encountered upon ocean entry.

We categorized the years into cold or warm ocean regimes by ranking the average value of the spring PDO from the long term data set of 1900–2011 and the coldest or most negative PDO years in our time series were grouped together to embody the “cold ocean regime” which included 1982, 1999–2002, 2007–2009, and 2011. The warmest or most positive PDO years, which represented the “warm ocean regime” were 1981, 1983–5, 1998, 2003–2006, and 2010. We used PDO instead of local measures of temperature as larger scale measures of temperature (such as the PDO) are more significantly related to juvenile salmon than local measures [4].

Spring Chinook salmon typically leave the freshwater environment at age-1 in the spring, and return as adults to their natal river 2 years later. Counting the returns of adult salmon at Bonneville Dam (first dam on mainstem of the Columbia River), which has data throughout our time series, and lagging the counts back two years is our proxy measure of ocean survival [4]. The data are available at: (http://www.fpc.org/adultsalmon/adultqueries/Adult_Table_Species_AllHistoric.html).

### Diet composition, fullness, and salmon energetic intake

For trophic analysis, we analyzed up to 30 stomachs from each station. Stomach contents were identified to the lowest possible taxonomic category using a dissecting microscope. Prey were enumerated and weighed to the nearest 0.001 g [30,32]. Following Daly et al. [17], prey were grouped into 15 trophic categories: Pacific sand lance (*Ammodytes hexapterus*), Northern anchovy (*Engraulis mordax*), Clupeids, Cottids, flatfishes (Pleuronectids), Osmerids (*Allosmerus elongatus* and other unidentified smelts), Rockfishes (*Sebastes* spp.), “unidentified fish” (unidentified fish and other rare fish that made up < 5% of diet composition), Copepods, Decapods (*Cancer* spp. larvae and other decapod larvae), Euphausiids, Hyperiids, Insects, Pteropods, and an "other" category (Cephalopods, Cirripede larvae, gelatinous zooplankton, Mysids, non-Hyperiid Amphipods, and Polychaetes).

We evaluated changes in salmon diet composition by weight of prey eaten for May and June for each year using Principal Coordinate Analysis (PCO). This method, which uses actual values rather than being rank distance based, attempts to maximize the variance preserved by particular ordination axes and best represent the spatial display of multivariate diet composition data. We also included environmental variables in a second matrix to relate to the diet composition structure. The diets were averaged for each month and year and matrices were evaluated based on the Bray-Curtis distance matrix and the environmental variables were normalized. May and June were evaluated separately. The environmental variables were correlated with the PCO axes and were visually displayed as vectors on the ordination plots. Axis values represent the annual separation or differences in diet composition and denote a univariate value of the interannual variability observed in the diet composition.

In order to evaluate if the diet composition varied significantly between ocean regimes, we tested for differences using the analysis of similarities (ANOSIM) test. The analysis is a multivariate analog to the analysis of variance (ANOVA) and was done at a finer spatial scale by using averaged diet composition for each sampling station where there were a minimum of 3 salmon with food [20,32]. The diet composition by weight of prey consumed were fourth-root transformed, and the analysis was based on Bray-Curtis matrices. May and June were evaluated separately. Lastly, to identify the prey categories which contributed to any significant regime differences, we utilized the similarity of percent contribution (SIMPER) analysis. PCO, ANOSIM, and SIMPER analysis were carried out using PRIMER6+ software [35].

Stomach fullness of the juvenile salmon was calculated as a percentage of their body weight (% BW) based on the follow equation:

$$\%\;BW\;=\;\frac{stomach\;content\;weight}{total\;fish\;weight\;-stomach\;content\;weight}\times 100$$

There was a significant negative correlation between length of the juvenile salmon and their stomach fullness, as smaller fish have larger stomach volume for their body length than larger fish, and as such have the potential for higher stomach fullness due to their size. In years when fish were significantly smaller, the fish could exhibit higher fullness due in part to their larger stomach volume relative their fork length [18]. In order to correct for the potential size bias in the time series for both the visual representation of stomach fullness, as well as the average interannual stomach fullness value used in general additive model analysis (GAMs; see below), we calculated an *index of stomach fullness*. Stomach fullness-length residuals were calculated by regression analysis of the ln(BW proportion + 0.01) with ln(fork length). The regression for May (n = 2019) and June (n = 1719) were determined separately. Negative values indicated that the juvenile salmon stomachs were less full for their fork length than would be expected. The average month/year index of stomach fullness was used for visual representations of fullness which incorporates the size of the predator and the GAM analysis. To test for statistical differences in stomach fullness for each month and ocean regime, we utilized a multifactor analysis of covariance (ANCOVA) with fullness as the dependent variable, fork length as the covariate, and regime as the factor. Relationships were considered significant at P < 0.05.

As prey types can have dissimilar energy densities, we wanted to identify if the amount of prey energy consumed during variable ocean regimes were different. As such, we converted the wet weight of the prey consumed per (g) of salmon to a standardized prey energetic density (kJ g^-1^). Literature values for prey energy content were utilized Pacific sand lance [36], Northern anchovy [37], Clupeids [38], Cottids [39], Flatfishes [38], Osmerids [38] Rockfishes [40], Energy values for unidentified fish consumed were the average kJ of known salmon fish prey for each month/year. Copepods [39], Decapods [41], Euphausiids [39], Hyperiids [39], Insects [42], and Pteropods [39]. Energy values for “other” invertebrates prey consumed were the average kJ of Cephalopods [40], gelatinous zooplankton [40], non-Hyperiid Amphipods [40], and Polychaetes [43]. The standardized energetic densities for each regime and month were tested for differences using the Mann-Whitney U test. This test was chosen for the statistical analysis due to the non-normality of the data and significance was set at P < 0.05 for all tests.

### Salmon condition metrics

Juvenile salmon size and length-weight condition factor were considered indicators of salmon growth. The condition factor index was calculated for individual fish based on ln(weight) to ln(length) residuals from regression analysis. A positive condition factor would indicate that the juvenile salmon were heavier than would be expected given their fork length. May (n = 3378) and June (n = 2344) regressions were calculated separately. Condition factor length-weight residuals were averaged for each month/year for a visual representation. Statistical analysis of the differences between salmon fitness metrics (length and condition) and ocean regime were tested using the Mann-Whitney U test.

### General additive models

We used generalized additive models (GAMs) to determine if variation in biological metrics of juvenile spring Chinook salmon were related to climate indices. Six different salmon biological metrics for both May (15 years) and June (19 years) were modelled separately using GAMs with the following as the interannual dependent variables; the diet composition as measured by both x and y axis of the PCO, average of the index of stomach fullness, average standardized energy consumed, average condition factor, and returns of adult spring Chinook salmon as a proxy for survival. We incorporated a number of climate indices as explanatory variables after first examining correlation among the variables. Climate indices utilized were SST, EET, PDO, UPW, COR, and NPGO. Correlated variables were not fitted in individual models together (ie. PDO, SST, NPGO, or UPW and EET), but models were run separately. GAMs were constructed with the R package, *mgcv* (version 1.7–22; [44]) using the Gaussian family with an identity–link function. Explanatory variables were modeled as continuous variables and smoothed. We adopted the “backward-selection” model selection strategy by starting with a full model, and subsequently dropping insignificant explanatory variables to arrive at the final model. Candidate predictors that were statistically significant at the P = 0.05 level were retained during the selection process. We selected the final model by minimizing the Generalized Cross Validation (GCV) scores.

## Results

From the two sampling periods, there were 3383 yearling juvenile Chinook salmon collected during May surveys and 2351 from June surveys, and trophic analysis was completed for 1863 May sampled salmon and 1653 June sampled fish (S2 Table). Chinook salmon preyed primarily on juvenile fishes during May (71.8 ± 39.2% fish prey) and June (73.6 ± 38.9% fish prey). Highest average piscivory rates were observed in the May warm ocean regime (79.9 ± 34.9%) and piscivory rates were significantly lower in cold May regimes (66.9 ± 41.0%; Mann-Whitney; P < 0.0001). Piscivory rates in June were also significantly higher during the warm ocean regime (75.5± 38.4.0% versus 72.4 ± 39.1%; Mann-Whitney; P = 0.03).

### Diet composition

**Fig 2:**
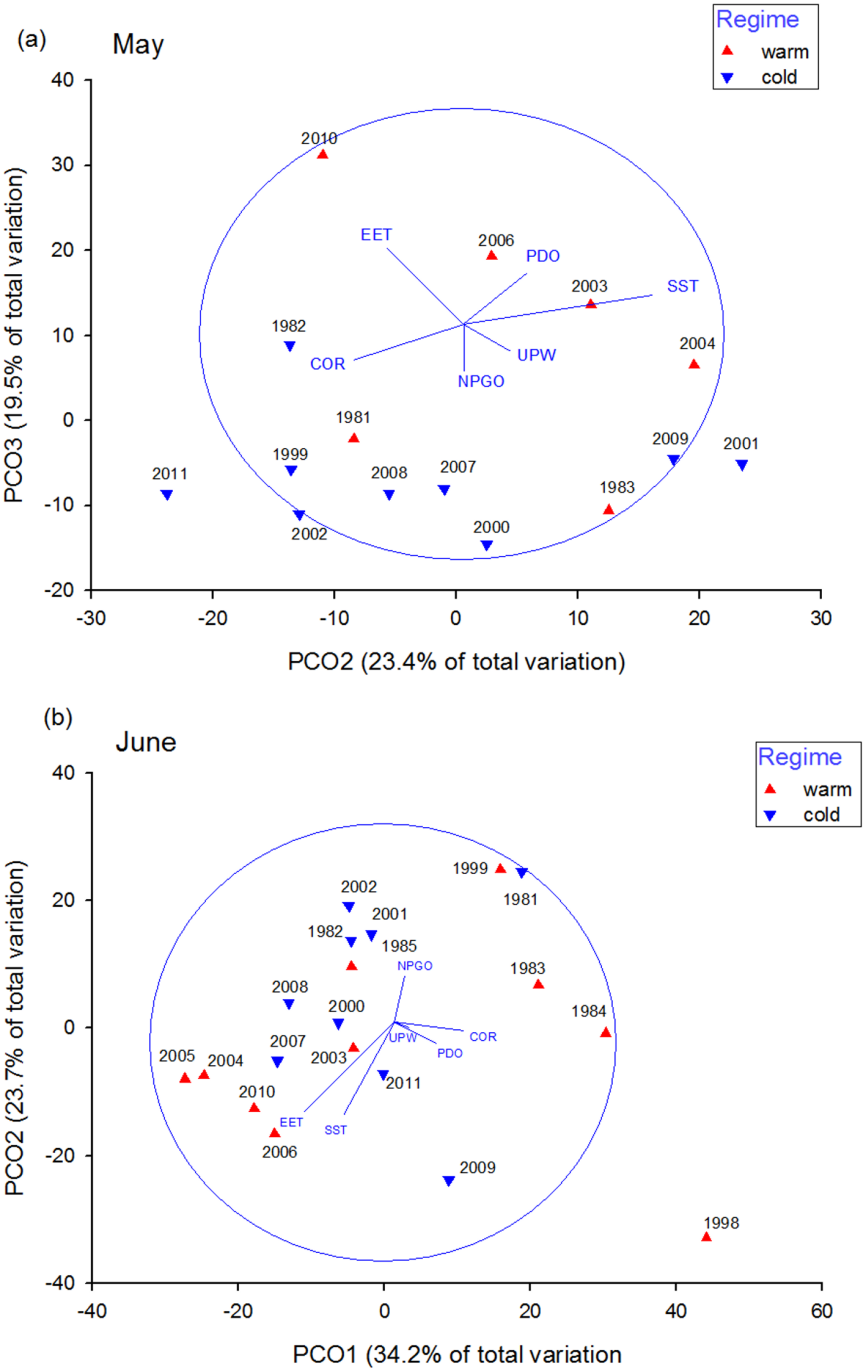
Principal Coordinate Analysis (PCO) of juvenile Chinook salmon diet composition in May (a) and June (b). Each data point on the PCO plot represents the average annual diet composition of juvenile Chinook salmon for May and June. The vectors show the direction and relative strength of the environmental variables examined and the circle represents equilibrium.

The diet composition of juvenile Chinook salmon in May and June varied annually, with cold and warm ocean regime diet composition grouping separately from each other (Fig 2). In May, most warm ocean regime years were positively oriented to the x and y axis of the PCO along with positive values of temperature (PDO and SST), and lower spring Columbia River flow rates (COR; Fig 2a). In 2010, diet composition displayed the most extreme placement in ordination space the most extreme placement in ordination space (Fig 2a). Cold years (except 2001 and 2009) were oriented negatively along the x and y axis. The June diet composition separated by regime with most of the cold ocean regime clustered in the center of the plot, except 1999, and warm ocean regime years on both the positive (1980’s warm years; 1998) and negative 2004–2006; 2010) areas of the x axis (Fig 2b). June diet composition of 2004–2006 and 2010 were grouped together as warm and low Colombia River flow years (COR). The most extreme outlier year was 1998 (Fig 2b).

In May, diet composition differences were overall significant by the factor of regime (S2 Table; ANOSIM; Global R = 0.122; P = 0.001). During May, there were significantly higher proportions of euphausiids (primarily *Thysanoessa spinifera*) and Pacific sand lance in cold ocean regimes, and more rockfish, decapod larvae (primarily *Cancer* spp. megalopae), and flatfish in warm ocean regimes, (based on SIMPER analysis). These six prey taxa contributed to 51.5% of the significant differences in the warm versus cold. In June, diet composition were also significantly different in cold versus warm ocean regimes (S2 Table; ANOSIM; R = 0.074; P = 0.001). Higher amounts of rockfish and decapod larvae were eaten in the warm ocean regime, and more “other” prey, and euphausiids were eaten during cold ocean conditions, and in contradiction to May diet composition differences, more decapods and Cottids in cold ocean regime (SIMPER). These five prey taxa contributed to 56.3% of the significant differences in the warm versus cold ocean regimes. Overall, the taxa most responsible for regime diet composition differences were changes in the amount of juvenile rockfishes eaten in both May and June.

### Stomach fullness

**Fig 3:**
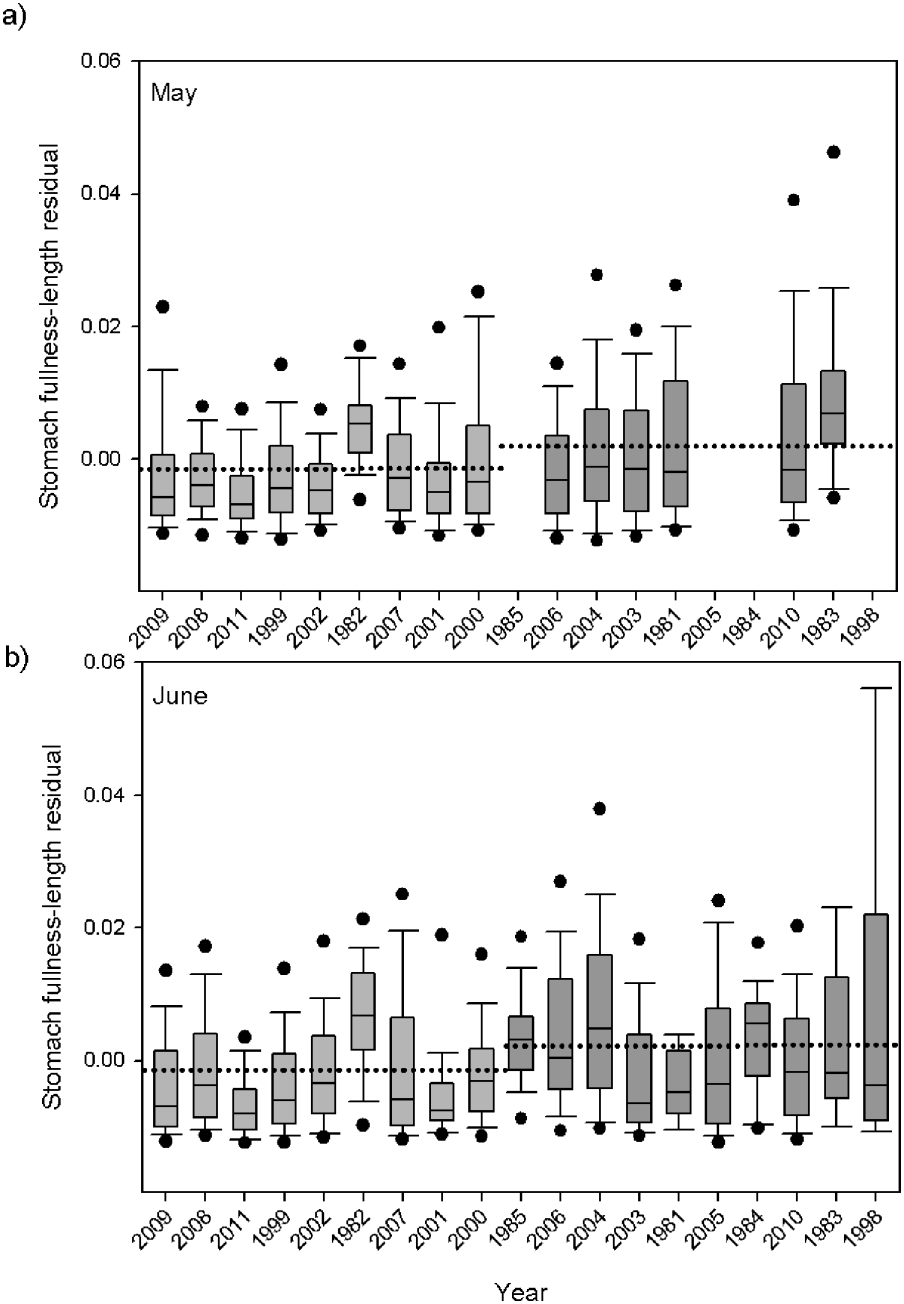
Average annual index of stomach fullness for May (a) and June (b) of juvenile yearling Chinook salmon. Shown are boxplots with the median (lines in boxes), 25th and 75th percentiles (lower and upper ends of boxes), 10th and 90th percentiles (error bars), and 5th and 95th percentiles (points) for each year. Light grey bars designate cold years, and darker grey bars designate warm years, with dotted line representing regime median.

Juvenile Chinook salmon had higher stomach fullness during the warmer than the colder ocean regime for both study months (Fig 3). Juvenile salmon in the May warm ocean regime consumed on average 1.3 (± 1.2)% of their body weight, whereas in the cold ocean regime, they were eating prey amounts equivalent to 0.9 (± 0.9)% of their body weight. Juvenile Chinook salmon in the May warm ocean regime had significantly fuller stomachs than during the cold ocean regime fish (ANCOVA; P < 0.0001). During May, juvenile salmon in the length range of 145–165 mm consumed approximately 28% more grams of food during the warm ocean regime. In June, salmon had eaten an average prey amount of 1.0 (± 1.0)% of their body weight in the cold ocean regime, and 1.4 (± 1.3)% in the warm ocean regime. This translates to a typical salmon in June the length range of 145–165 mm eating 30.5% more food during the warm ocean regime. Salmon in June had significantly fuller stomachs in warm ocean regime conditions than cold (ANCOVA; P < 0.0001). In both May and June, more salmon had empty stomachs in the cold ocean regime. In May, 7.9% and 5.45% of stomachs were empty and 8.1%, 7.4% were empty in June during cold/ warm ocean regimes respectively.

### Salmon energetic intake

**Fig 4:**
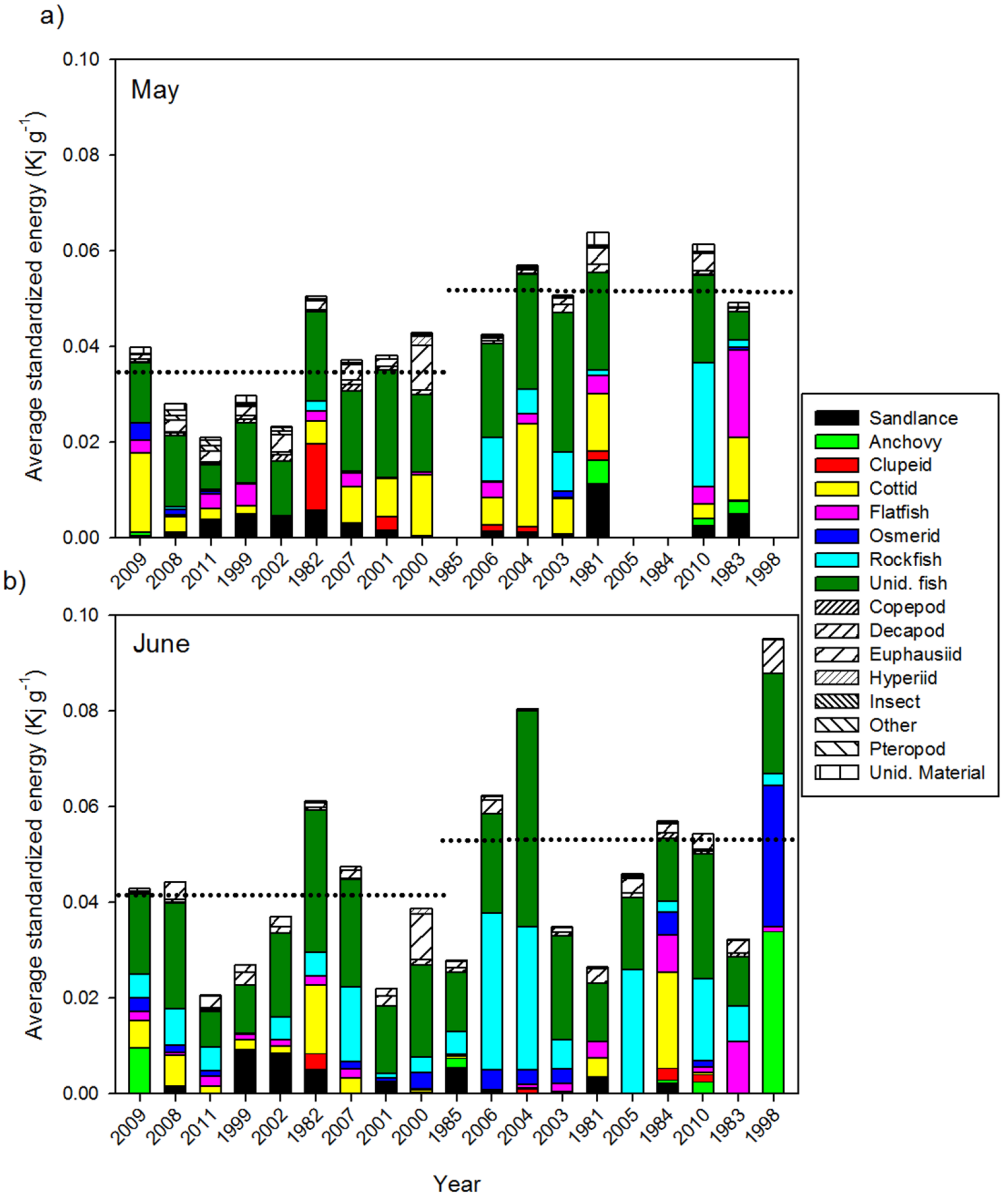
Average standardized prey energy density eaten (kJ g^-1^) by sampling year and May (a) and June (b). The x-axis years are ranked by average spring Pacific Decadal Oscillation (low to high) with cold regime on left of vertical dashed line, and warm regime to the right and with horizontal dotted line representing regime median.

Juvenile salmon consumed different prey types, had different stomach fullness, and also had a difference in total amount of prey energetic density eaten between ocean regimes. Prey taxa of highest energy density were anchovy and clupeid fish, and the lowest energy were copepods, pteropods and hyperiid amphipods. The amount of energy consumed per predator indicated that juvenile Chinook salmon consumed higher amounts of energy during the warmer ocean regime (Fig 4). On average, salmon in the cold May ocean regime consumed 0.035 (± 0.037) kJ g^-1^of prey, and in the warm regime, they consumed 0.052 (± 0.048) kJ g^-1^. These differences were significant, with 33% less total energy being consumed during cold ocean conditions in May (Mann-Whitney; P < 0.0001). In June, juvenile Chinook salmon consumed significantly lower total energy per gram during cold (0.041 ± 0.041 kJ g^-1^) than warm (0.053 ± 0.052 kJ g^-1^) ocean conditions (Mann-Whitney; P < 0.0001). Salmon in June consumed on average 23.1% more kJ g^-1^ during the warm ocean regime.

### Salmon condition metrics

**Fig 5:**
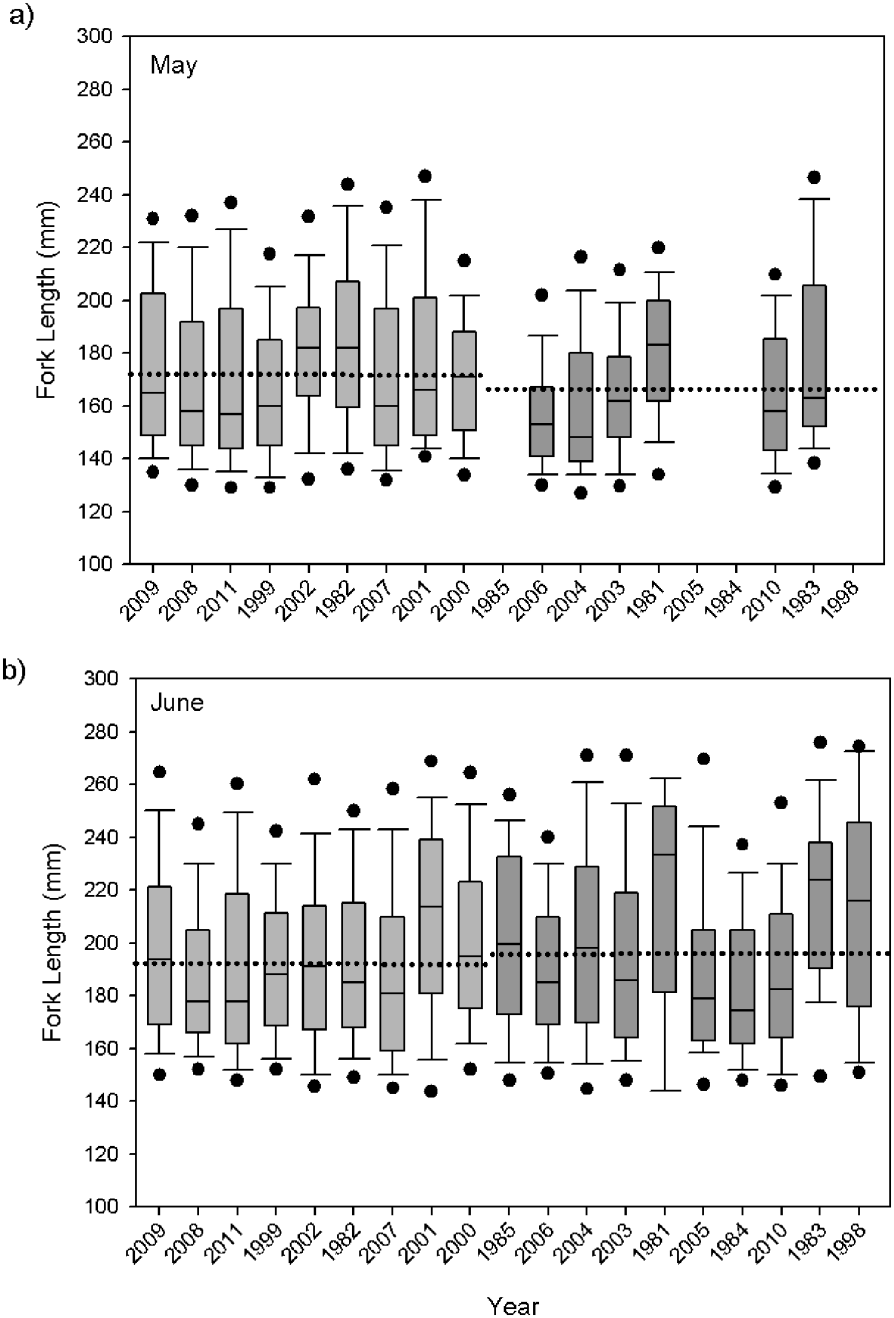
Average juvenile yearling Chinook salmon annual fork length (mm) for May (a) and June (b). The x-axis years are ranked by average spring Pacific Decadal Oscillation (low to high). Shown are boxplots with the median (lines in boxes), 25th and 75th percentiles (lower and upper ends of boxes), 10th and 90th percentiles (error bars), and 5th and 95th percentiles (points) for each year. Light grey bars designate cold years, and darker grey bars designate warm years with dotted line representing regime median.

**Fig 6:**
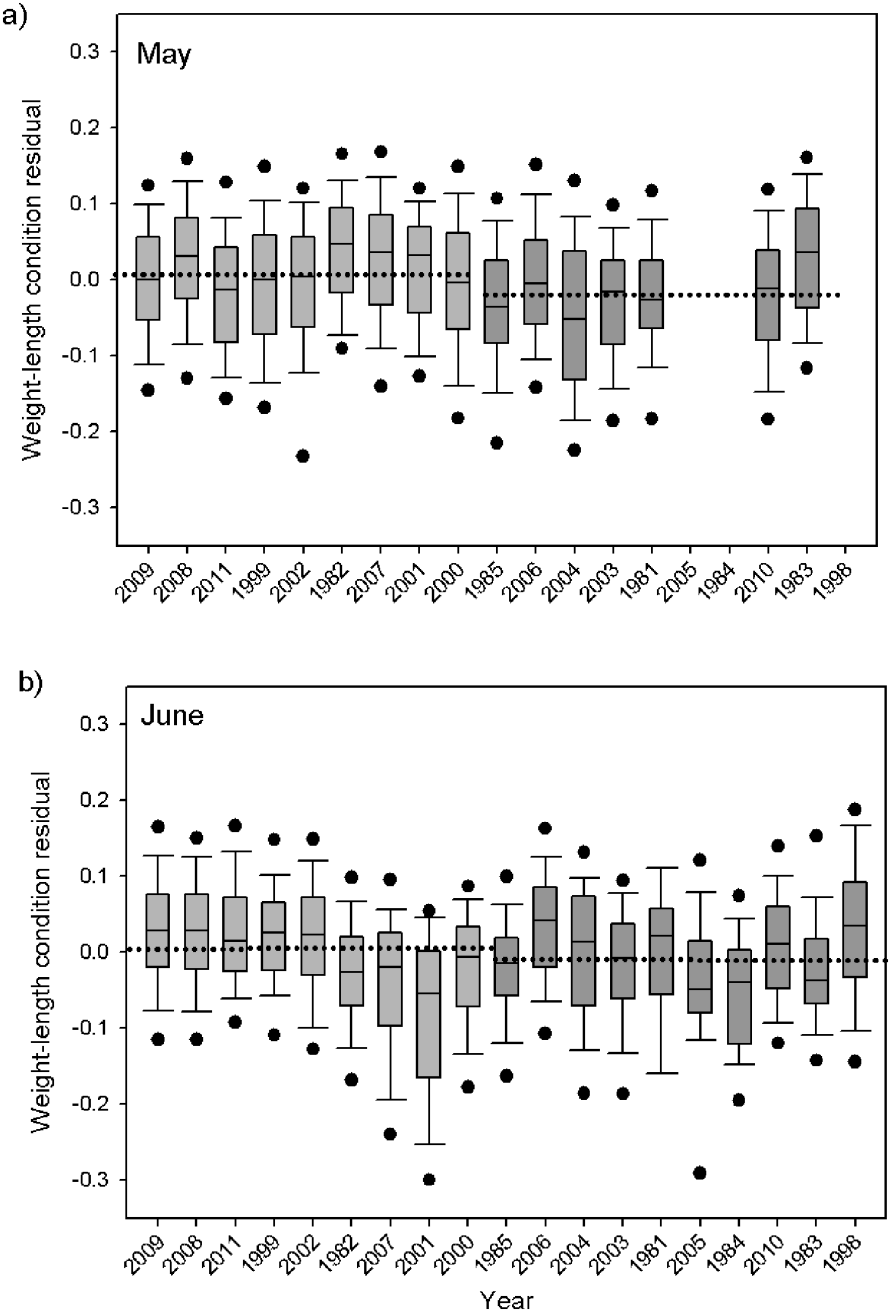
Average juvenile yearling Chinook salmon annual weight-length condition residual for May (a) and June (b). The x-axis years are ranked by average spring Pacific Decadal Oscillation (low to high). Shown are boxplots with the median (lines in boxes), 25th and 75th percentiles (lower and upper ends of boxes), 10th and 90th percentiles (error bars), and 5th and 95th percentiles (points) for each year. Light grey bars designate cold years, and darker grey bars designate warm years with dotted line representing regime median.

The size of the juvenile Chinook salmon also differed between ocean regimes. Salmon were significantly longer during the May cold ocean regime than warm (Mann-Whitney; P < 0.0001; Fig 5), as well as heavier for their size. In May, the average length in the colder regime was 171.9 (± 31.3) mm and the fish weighted on average 62.4 (± 40.8) g, whereas the average size during the warm ocean regime was smaller at166.2 (± 27.8) mm and thinner at 53.1 (± 33.0) g. The condition of the fish based on length-weight residuals in the cold May ocean regime was significantly greater than those from the warm regime (Mann-Whitney; P < 0.0001; Fig 6). In June, salmon were significantly shorter during the cold ocean regimes (192.1 ± 32.2 mm; Mann-Whitney; P = 0.016) and weighted on average 92.3 (± 54.3) g, while fish in the warmer regimes were on average 196.1 (±34.8) mm and 98.8 (± 59.8) g. Though the fish were smaller in the cold regime, they had significantly better condition–or fatter for their size than in warm years (Mann-Whitney; P < 0.0001; Fig 6).

### Relationship to climate indices

Ocean climate indices of temperature (PDO, SST or NPGO), Eastern Ekman transport (EET), upwelling (UPW), and Columbia River flow (COR) were significantly related to Chinook salmon biological indices based on general additive models (GAMs). In both May and June, average annual index of stomach fullness and salmon energetic intake were positively related to increased temperatures, and both salmon condition factor and adult returns of juvenile salmon were negatively related to metrics of temperature (except NPGO with positive values equal to colder ocean conditions; Tables 2 and 3; S1–S3 Figs). The condition and adult returns of juvenile salmon were positively related to increases in spring river flow (COR) in May. In May, four out of the six models had explained greater than 75% of the variability in the dependent variable (R^2^ from 0.75 to 0.92; Table 2; S1 Fig). In June, the GAMs were less explanatory with the exception of survival of Chinook salmon based on adult return counts (70.4% deviance explained; Table 3; S2 Fig). In summary, increased adult counts of Chinook salmon (ocean survival) related linearly to juvenile salmon first marine summer ocean conditions of colder temperatures, increased Columbia River flow (March-May), higher fish condition measures, and non-linearly to prey energy density amounts (S3 Fig).

**Table 2:** General additive models of May salmon biological and environmental variables.

Variable	Best-fit Model Environmental Variables			R^2^	DEV
**PCO2**	SST*(+)			0.35	40.0%
**PCO3**	COR**(-)	NPGO**(-)	EET*(+)	0.65	78.6%
**FEED**	COR***(-/+)	SST* (+/-)	EET**(+/-)	0.88	95.3%
**PREY**	PDO*** (-/+)			0.67	73.9%
**COND**	COR***(+)	EET***(-)		0.75	81.8%
**RETURN**	COR*(+)	NPGO***(+)	PREY**(+/-)	0.92	96.3%

Best-fit GAM models in May with R^2^ and deviance explained (DEV) for the relationships between the first two diet composition PCO axes (PCO2, PCO3), index of stomach fullness (FEED), prey caloric density (PREY), condition (COND), and counts of spring Chinook salmon at Bonneville Dam (RETURN) of juvenile Chinook salmon and environmental variables (i.e., COR = Columbia River Flow, PDO = Pacific Decadal Oscillation, NPGO = North Pacific Gyre Oscillation, SST = sea surface temperature, and EET = Eastern Ekman Transport). Significance levels of the variables are denoted with asterisks:

* = p < 0.05,

** = p < 0.01,

*** = p < 0.001.

The type of effect from each of the model variables on salmon biological metrics is in parentheses (i.e., + = positive,— = negative, -/+ = negative followed by positive nonlinear, +/- = positive followed by negative nonlinear, etc.).

**Table 3:** General additive models of June salmon biological and environmental variables.

Variable	Best-fit Model Environmental Variables			R^2^	DEV
**PCO1**	COR*(+)	EET* (-)		0.27	39.4%
**PCO2**	No significant relationship				
**FEED**	COR*(+)	PDO** (+)	UPW** (+)	0.4	50.3%
**PREY**	SST* (+)			0.23	28.2%
**COND**	No significant relationship				
**RETURN**	NPGO***(+)	COND*(+)		0.64	70.4%

Best-fit GAM models in June with R^2^ and deviance explained (DEV) for the relationships between the first two diet composition PCO axes (PCO1, PCO2), index of stomach fullness (FEED), prey caloric density (PREY), condition (COND), and counts of spring Chinook salmon at Bonneville Dam (RETURN) of juvenile Chinook salmon and environmental variables (i.e., COR = Columbia River Flow, PDO = Pacific Decadal Oscillation, NPGO = North Pacific Gyre Oscillation, SST = sea surface temperature, UPW = upwelling, and EET = Eastern Ekman Transport). Significance levels of the variables are denoted with asterisks:

* = p < 0.05,

** = p < 0.01,

*** = p < 0.001.

The type of effect from each of the model variables on salmon biological metrics is in parentheses (i.e., + = positive,— = negative, -/+ = negative followed by positive nonlinear, +/- = positive followed by negative nonlinear, etc.).

## Discussion

In the present study we determined that during warm ocean regimes, juvenile spring Chinook salmon consumed different prey types, had significantly fuller stomachs, consumed more prey energy, were in poorer condition, and had lower return numbers as adults. With oceans predicted to warm due to global climate change, we expect Pacific salmon in the California Current to be negatively impacted [45]. For ectotherms, temperature is the ‘abiotic master factor’ [46] and climate-atmospheric linkages of decreased survival during warmer ocean conditions have been previously shown for multiple North Pacific Ocean salmon species. During warmer ocean regimes, or more positive Pacific Decadal Oscillation phases, adult return numbers decrease compared to cooler phases [2,4,47]. Linking a less productive and warmer ocean environment with decreased survival suggests that during warmer ocean regimes, lower prey resources are available and fish grow slower and remain for a longer period at the size that makes them vulnerable to size-limited predation. In contrast, during colder ocean regimes with higher ocean productivity, the surplus availability of food would not limit growth and thus decrease the time that the fish are vulnerable to ocean predators. The well-established relationship of decreased salmon production during warm ocean regimes is primarily focused on decreased food resources which lead to decreased growth and survival. Our long term data set contradicts the assertion that juvenile salmon eat less in warm regimes, as the fish have significantly fuller stomachs and more calorie equivalents in warm ocean regimes, and they exhibit lower condition and adult returns than in colder regimes.

In addition to ocean temperature, Columbia River flow was also linked to spring Chinook salmon diet composition changes, stomach fullness, condition and returns of adult salmon two years later, but the effect that river flow or the size of the ocean river plume has on salmon ocean ecology is less well understood relative to the PDO. Miller et al. [47] observed that the Columbia River plume size was related to salmon adult returns only during years of moderate ocean conditions, as during poor ocean conditions, the river plume minimally related to adult returns. Our study identified that during increased spring Columbia River flow, significant changes in diet composition, and increased condition and return numbers occurred, as well as a significant non-linear relationship to stomach fullness. Prey changes in and around the plume have been documented under differing ocean conditions [48–50], but how variability in the plume affects the production or type of prey community suitable for juvenile salmon is unknown. Higher plume volume has been linked to better steelhead survival, but the mechanism is unclear [51]. Chinook salmon diet composition, stomach fullness, condition, and returns were also affected by environmental factors of transport as measured by upwelling and Ekman transport which has been shown to relate to salmon [3].

When individual fish forage for food, their uptake of energetic and material resources is converted in their body, and then allocated to fitness-enhancing processes of survival and growth and ultimately reproduction [52]. As ectotherms, salmon are temperature dependent [22,27,28], and if food rations are lower than optimal, then the optimal temperature for salmon growth is lower [27,53]. For our study, the average SST differed by less than 3°C between cold and warm ocean regimes. Even with this relatively small temperature increase, salmon had fuller stomachs, yet gained less body weight and had lower returns as adults. While growth rates under warmer water conditions can increase if there is sufficient food resources, unless the temperature is too high, the food intake and evacuated rate also increases thus decreasing the ability of the fish to convert their food to somatic growth [24,28,29]. Research on salmon in freshwater has shown that higher stream temperatures result in a higher metabolic cost that requires more food to maintain similar growth rates as found in cooler streams [54].

Prey resources did not appear to be at sufficient levels for juvenile salmon during warm ocean regimes as indicated by juvenile spring Chinook salmon growing and surviving less successfully. Daly et al. [17], using a long time series, provided evidence that prey fish biomass available to juvenile salmon was significantly lower during warmer ocean conditions, and lower prey biomass levels have been shown to explain reductions in returns of adult salmon [17,55]. Other important prey eaten by juvenile salmon with long term abundance data are juvenile ocean shrimp *(Pandalus jordani)* and Dungeness crab megalopae *(Cancer magister)* and both reflect lower abundance during increased SST [56,57]. The copepod community from the Newport Hydrographic Line (latitude 44.65°N) also show lower biomass of copepods during warmer ocean regimes, which was related to a reduction in the number of adult salmon returns [4]. Since spring Chinook salmon rarely consume copepods [30,32], this relationship may reflect the decreased feeding success during warmer ocean regimes of the dominant prey of juvenile salmon (i.e., juvenile fishes) which feed directly upon copepods. Prey resources in warm ocean regimes appear to be lower than in cold years, and we therefore would expect to see higher numbers of empty stomachs and lower stomach fullness, yet our results show the opposite pattern. More prey may need to be eaten in warmer ocean regimes for several reasons; 1) physiologically they require more food due to temperature dependent increases in metabolism, 2) with reduced prey, the salmon have higher foraging energy expenditures, and/or 3) the energetic quality of prey in warmer years is reduced and as such, the salmon need to eat more. An alternate reason for more food being consumed during the warmer ocean regimes may be that the salmon in the warm ocean years represent the best foragers within the population, while the salmon less able to capture prey during reduced resources have already been consumed by predators.

Another diet change exhibited by juvenile salmon during warmer ocean regimes was that they consumed more prey from higher trophic levels by exhibiting greater piscivory rates, especially during El Niño years [58], yet this better-quality trophic level diet did not correspond to a healthier body condition. This inconsistent relationship was also found in a threatened seabird species [59]. During cold ocean years, there is evidence that there are more prey biomass available to juvenile salmon and this “surplus” of food translated to higher salmon growth and return as adults [4], yet the salmon are eating lower quality prey (less piscivorous) and less food during the higher prey biomass years. Increased growth observed while the juvenile salmon consumed less food biomass and food of lower energetic quantity could be in part related to the decreased energetic cost of foraging under conditions of plentiful prey as well as an increased ease of capturing less mobile prey of lower trophic levels.

The increased metabolic demand of juvenile salmon under warmer water temperatures along with lower food resources may interact to influence trade-offs between salmon foraging and predator avoidance. Actively hunting for more food would require juvenile salmon to increasingly focus on feeding behaviors versus predator avoidance, thereby increasing their risk of being consumed by predators. Risk assessment of ectotherms, when exposed to a threat, has been shown under laboratory conditions to change under variable temperatures and food ration levels [60]. Fish maintained at both warmer and cooler temperatures and high food rations exhibited anti-predator responses and decreased their foraging efforts. But the fish kept in 3°C warmer water, with low food rations, fed at a high rate even under the threat of predation [60]. In a field situation, Biro et al. [61] identified that age-0 trout in low-food lakes increased risk-taking behaviors to grow at maximum growth rates, and were at higher risk for predation. Juvenile salmon may also feed at a higher rate in low food and/or warmer ocean regimes regardless of predator presence, which could be reflected in population declines seen in warmer ocean regimes.

The metabolic demand on fish is both temperature and size dependent, and the optimal temperature for food conversion efficiency is higher for smaller fish [28]. With the majority of spring Chinook salmon being of hatchery origin which are significantly larger than fish of natural origin [32], hatchery managers may have increased success of their fish if they release them at smaller sizes [21]. If hatchery fish were to be released at a smaller size, they may be better able to utilize the food resources available to them in warmer ocean regimes. The potential increased adult return rate of the smaller-sized naturally-produced fish [62] could in part be due to their size related metabolic advantages and warrants further study.

In addition to observed changes in the stomach fullness under warmer ocean regimes, we observed changes in the prey type being consumed. Diet composition changes in warmer ocean regimes may reflect some prey population increases and/or spatial habitat changes during warmer ocean waters making different prey available to the salmon. Juvenile rockfish (*Sebastes* spp.) are fish prey that are eaten in higher proportions during warmer ocean conditions. During the warm El Niño of 2010, juvenile rockfish were highly abundant in the environment [50,63], and were eaten at higher rates by juvenile salmon during May of that year (this study). Also, only during warm ocean regimes did rockfish appear in any significant amount in the diets of juvenile salmon in May. During these warmer ocean regimes, the prevailing winds, spring transition timing, and upwelling strength all differ from the colder ocean regimes and this is reflected in a more offshore fish community, resulting in rockfish being more available on the shelf where salmon forage [50, 64].

During warmer ocean regimes, the timing of when salmon prey become available in the environment may shift to earlier in the year, potentially creating a timing mismatch in food resources needed when salmon enter ocean waters in spring [65]. Northern anchovy have been shown to spawn several months earlier than normal off Oregon during El Niño years [63,66], thus becoming more available to be preyed upon by juvenile salmon in May and June during their early juvenile stage. The timing of egg production in Black Sea anchovies advanced 12 to 19 days earlier with every 2°C increase in SST [67]. However, juvenile salmon typically enter the ocean around the same time each year [68]. An earlier spawning of prey would lead to a longer developmental period and could result in individual prey that are too large to be consumed by early juvenile salmon, creating a potential reduction in suitable food resources during warmer regimes. Timing of hatchery releases of juvenile salmon may need to be adjusted earlier due to warming ocean regimes under climate change.

One potential issue within our study is that although the yearling Chinook salmon from the OSU and Plume study were sampled and compared during the same time of year and geographic area, we assumed that they are an interchangeable group of fish to measure biological changes affecting them due to ocean conditions. The OSU study fish were larger in fork length than the more recent study, which could be due to hatchery practice modifications, changes in the proportion of the fish that were of hatchery versus naturally produced, and/or changes in the timing of ocean entry over the last 15–20 years (see also [20]). Further analysis could be done to address questions of differences of the yearling Chinook salmon between these two studies.

A secondary issue is that our use of published literature values for the caloric value of the prey did not take into consideration potential interannual variability, which could potentially bias our results. Based on our observed changes in diet composition, spring Chinook salmon were consuming higher amounts of caloric energy during warm ocean regimes. Studies looking at interannual variability in caloric values are limited for the Northeast Pacific Ocean and we could only identify 2 studies that looked at interannual changes in energy density, but both studies identified report significant interannual differences [69,70]. Beaubier and Hipfner [70] looked at juvenile and adult forage fish energy values between two years, and of the fish that were significantly lower in total energy density, this change was due to a reduction in their lipid energy density. Anthony et al. [69] found that most of the forage fish species they looked at did not have interannual differences in energetic content during the two years they examined, but juvenile Pacific herring did show interannual changes and again the differences were due to changes in their lipid content. If the prey taxa consumed during warmer ocean regimes have lower energetic content due to decreases in their lipid levels, then our study would be overstating the differences between total energy eaten between cold and warm years. Other aspects (fatty acid composition) of the quality of the prey that salmon consume could also vary on an interannual basis [71, 72], and clearly more studies are needed on how this may affect salmon in the future. Rosa et al. [73] examined interannual changes in energy density and lipid levels in European sardines (*Sardina pilchardus*) in the upwelling region off of Portugal and found that energy density can vary by 20–30% and lipids by close to 100% between years depending on environmental conditions. Changes in quality of prey have been proposed as a mechanism leading to long-term declines in Atlantic salmon (*Salmo salar*) off the northeast coast of North America [74] where both changes in prey composition and decreases in energy density within dominant prey were observed [75]. Further research into interannual energetic changes of salmon prey would be important to fully understand this effect on salmon growth and survival under warming ocean conditions.

We have used a long term time series to predict what potential impact global climate change will have on Chinook salmon feeding, growth and returns as adults. There is uncertainly in how conditions in the California Current will change under global climate change and how these new conditions may affect salmon. Although sea surface temperatures in the California Current are generally projected to increase by several degrees over the next half century based on climate global climate modelling [8,76], the temperature response at smaller scales may be highly variable. In fact, the NCC has shown a slight cooling trend in the last couple of decades perhaps due to increased coastal upwelling resulting from greater land-sea pressure differences [77–80]. Moreover, fish production has been predicted to increase in the California Current in the coming century under some climate scenarios [81].

Our results suggest that if warmer temperatures become prevalent in the NCC, juvenile spring Chinook salmon may be unable to meet their early summer metabolic demand and will continue in their population decline. Our findings are similar to those observed for a long time series on the bioenergetics of steelhead which predict lower growth and population declines under warmer ocean temperatures [21]. More detailed bioenergetics models of this population of salmon may allow managers the ability to determine the effects of changing ocean conditions on NCC Chinook salmon juveniles [24,26].

## Conclusions

In summary, juvenile salmon risk-assessment during warmer ocean regimes may affect their choice of action due to the burden of their increased metabolic state. This metabolic burden would affect the entire population of spring Chinook salmon as they migrate to sea, and the expected global climate change scenario of increased surface ocean temperatures may negatively impact threatened and endangered salmon. Our results underscore the importance of long-term time series in order to understand and predict how climate change will affect salmon populations and their marine survival.

## Supporting Information

S1 Fig General additive models for May biological and environmental variables. Fitted lines (solid) and 95% confidence intervals (dotted) of the additive effects of significant relationships on May salmon characteristics from the best-fit GAM models. March- May average environmental variables: COL = Columbia River Flow, PDO = Pacific Decadal Oscillation, SST = sea surface temperature, and EET = Eastern Ekman Transport.
(TIF)

S2 Fig General additive models for June biological and environmental variables. Fitted lines (solid) and 95% confidence intervals (dotted) of the additive effects of significant relationships on June salmon characteristics from the best-fit GAM models. April- June average environmental variables: COL = Columbia River Flow, UPW = Coastal upwelling index, PDO = Pacific Decadal Oscillation, SST = sea surface temperature, and EET = Eastern Ekman Transport).
(TIF)

S3 Fig General additive models for adult salmon returns and environmental variables. Fitted lines (solid) and 95% confidence intervals (dotted) of the additive effects of significant relationships on adult salmon returns from the best-fit GAM models. Environmental variables for figure a: COL = March-May average Columbia River Flow, NPGO = March-May average North Pacific Gyre Oscillation, and Kj = May average salmon energy density. Environmental variables for figure b: NPGO = April- June average North Pacific Gyre Oscillation, and Cond = June average salmon condition residuals.
(TIF)

S1 Table Resources for environmental data. Web sites for sources of environmental data that were accessed for relationships to salmon feeding and condition.
(PDF)

S2 Table Diet composition of juvenile salmon and their average size. Juvenile yearling Chinook salmon fork length (FL) in mm, sample size (*N*) and average diets by percent weight of major prey categories eaten in May and June. Empty stomachs were not included in the total counts.
(PDF)
