# Supplementary figures and images for: Warming Ocean Conditions Relate to Increased Trophic Requirements of Threatened and Endangered Salmon
Source: PLoS One. 2015 Dec 16;10(12):e0144066. doi: 10.1371/journal.pone.0144066 (PMC4682959; doi:10.1371/journal.pone.0144066)

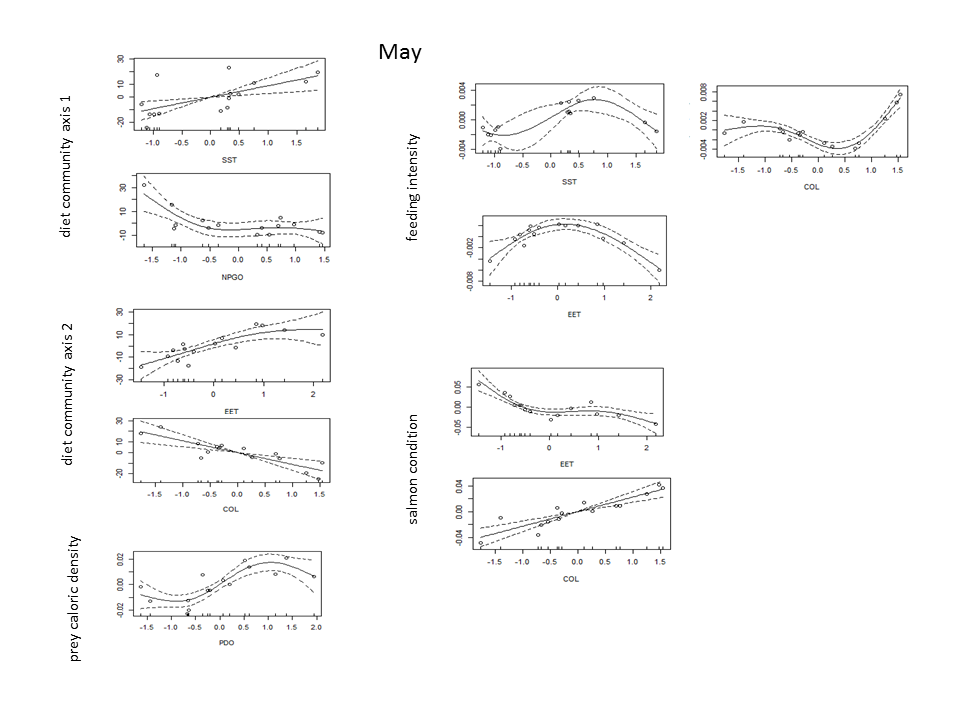

Supplement: S1 Fig — Fitted lines (solid) and 95% confidence intervals (dotted) of the additive effects of significant relationships on May salmon characteristics from the best-fit GAM models. March- May average environmental variables: COL = Columbia River Flow, PDO = Pacific Decadal Oscillation, SST = sea surface temperature, and EET = Eastern Ekman Transport. (TIF) [file pone.0144066.s001.tif]

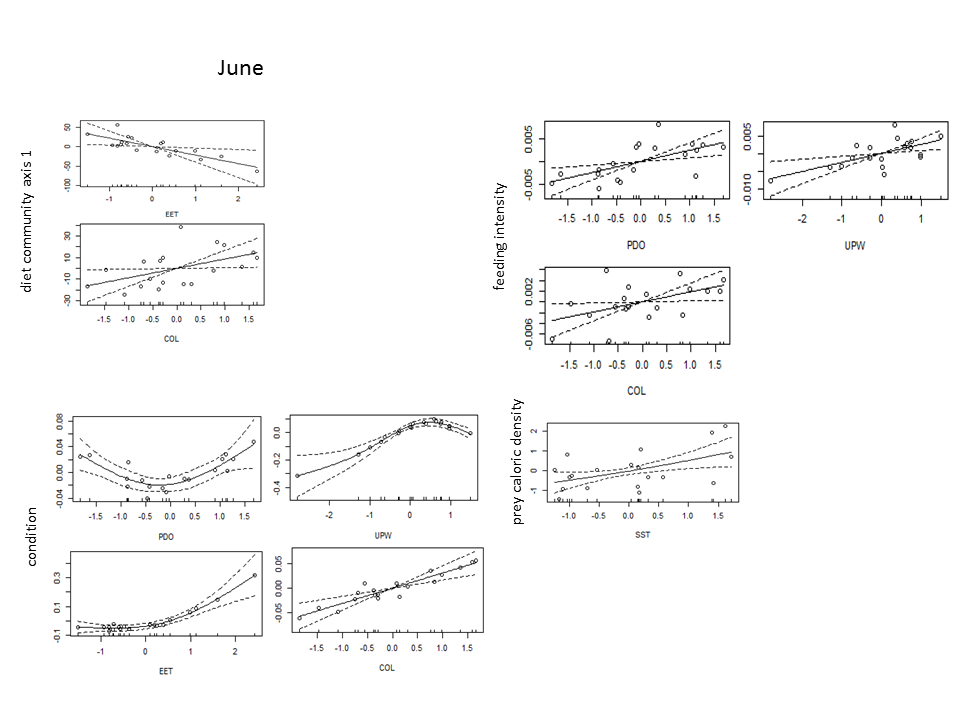

Supplement: S2 Fig — Fitted lines (solid) and 95% confidence intervals (dotted) of the additive effects of significant relationships on June salmon characteristics from the best-fit GAM models. April- June average environmental variables: COL = Columbia River Flow, UPW = Coastal upwelling index, PDO = Pacific Decadal Oscillation, SST = sea surface temperature, and EET = Eastern Ekman Transport). (TIF) [file pone.0144066.s002.tif]

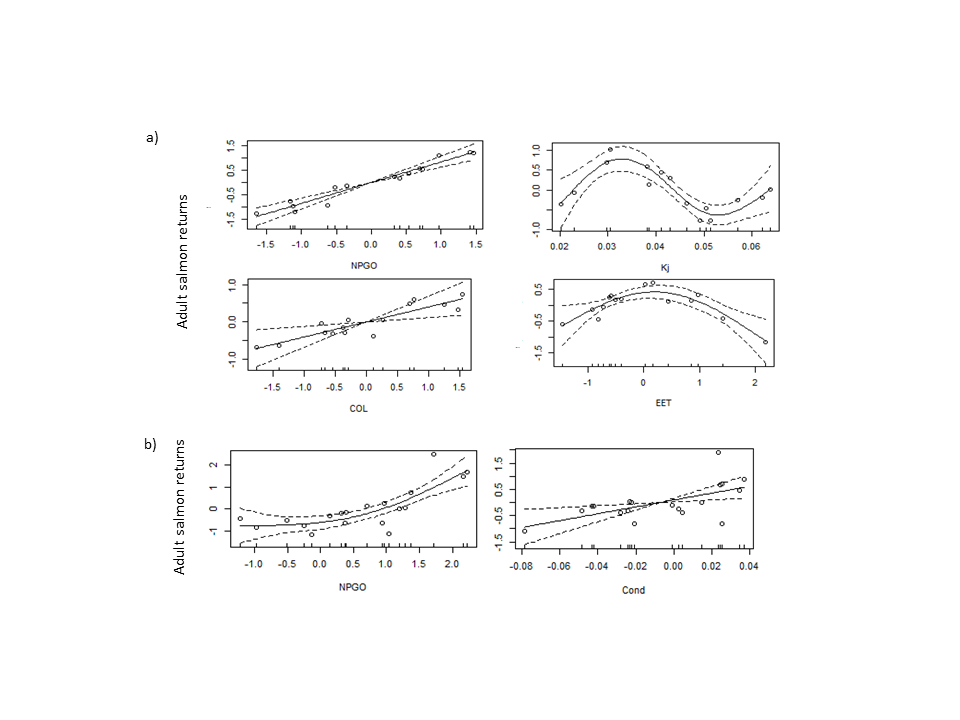

Supplement: S3 Fig — Fitted lines (solid) and 95% confidence intervals (dotted) of the additive effects of significant relationships on adult salmon returns from the best-fit GAM models. Environmental variables for figure a: COL = March-May average Columbia River Flow, NPGO = March-May average North Pacific Gyre Oscillation, and Kj = May average salmon energy density. Environmental variables for figure b: NPGO = April- June average North Pacific Gyre Oscillation, and Cond = June average salmon condition residuals. (TIF) [file pone.0144066.s003.tif]
