# Supplementary material for: Warming Ocean Conditions Relate to Increased Trophic Requirements of Threatened and Endangered Salmon
Source: PLoS One. 2015 Dec 16;10(12):e0144066. doi: 10.1371/journal.pone.0144066 (PMC4682959; doi:10.1371/journal.pone.0144066)
Supplement: S1 Table — Web sites for sources of environmental data that were accessed for relationships to salmon feeding and condition. (PDF) [file pone.0144066.s004.pdf]

Table S1. Resources for environmental data.

Sea Surface Temperature - SST (http://www.esrl.noaa.gov/psd/data/timeseries),

Pacific Decadal Oscillation - PDO (http://jisao.washington.edu/pdo),

North Pacific Gyre Oscillation - NPGO (http://www.o3d.org/npgo/npgo.php),

Coastal Upwelling Index - UPW (http://www.pfeg.noaa.gov/products/las.html),

Eastward Eckman Transport – EET (http://www.pfeg.noaa.gov/products/las.html)

Columbia River Flow at Bonneville Forebay - COR (http://www.cbr.washington.edu/dart/river.html).
