# Supplementary material for: Warming Ocean Conditions Relate to Increased Trophic Requirements of Threatened and Endangered Salmon
Source: PLoS One. 2015 Dec 16;10(12):e0144066. doi: 10.1371/journal.pone.0144066 (PMC4682959; doi:10.1371/journal.pone.0144066)
Supplement: S2 Table — Juvenile yearling Chinook salmon fork length (FL) in mm, sample size (N) and average diets by percent weight of major prey categories eaten in May and June. Empty stomachs were not included in the total counts. (PDF) [file pone.0144066.s005.pdf]

Table S2. Diet composition of juvenile salmon and their average size.

				Ammodytes	Anchovy	Clupeid	Cottid	Flatfish	Osmerid	Sebastes	Unid. Fish	Copepod	Decapod	Euphausiid	Hyperiid	Insect	Other	Pteropod
May		FL	*N* =															
	1981	179	**59**	26.2%	3.4%	2.4%	11.7%	9.5%	0.0%	2.0%	34.6%	0.0%	3.4%	4.9%	0.1%	0.0%	0.9%	0.9%
	1982	184	**86**	18.8%	0.0%	22.3%	9.5%	7.1%	0.0%	6.9%	28.5%	0.0%	1.0%	5.0%	0.2%	0.0%	0.4%	0.4%
	1983	185	**79**	14.0%	2.0%	0.4%	27.1%	34.5%	2.4%	2.9%	11.7%	0.1%	3.2%	1.6%	0.1%	0.0%	0.0%	0.0%
	1999	167	**160**	18.7%	0.0%	0.0%	5.1%	16.2%	0.3%	0.1%	43.3%	3.2%	2.0%	6.5%	1.3%	0.1%	2.3%	1.0%
	2000	175	**92**	1.1%	0.0%	0.0%	15.0%	1.0%	0.0%	0.1%	35.8%	0.1%	4.4%	29.5%	5.9%	0.0%	6.4%	0.7%
	2001	179	**72**	3.9%	0.0%	3.1%	30.1%	0.1%	0.0%	0.0%	51.3%	0.0%	5.4%	5.9%	0.1%	0.0%	0.1%	0.0%
	2002	180	**93**	11.5%	0.0%	0.6%	0.9%	2.1%	0.0%	0.5%	45.8%	3.8%	2.0%	24.4%	0.4%	0.0%	2.8%	5.1%
	2003	168	**109**	2.6%	0.0%	0.0%	9.0%	1.8%	9.3%	12.7%	54.1%	0.0%	5.1%	4.4%	0.3%	0.0%	0.8%	0.0%
	2004	168	**79**	4.3%	0.0%	2.3%	26.4%	4.0%	0.0%	11.5%	45.3%	0.7%	2.6%	0.7%	0.0%	0.0%	0.4%	1.7%
	2006	159	**236**	5.4%	0.0%	1.7%	10.5%	8.4%	0.3%	21.7%	45.8%	0.1%	1.6%	1.4%	1.6%	0.0%	1.4%	0.1%
	2007	163	**166**	7.2%	0.0%	0.0%	15.9%	8.1%	0.1%	0.9%	41.8%	4.5%	2.5%	14.2%	0.9%	0.0%	3.0%	0.9%
	2008	189	**178**	5.0%	0.0%	0.2%	9.3%	1.3%	1.9%	1.8%	41.5%	2.0%	1.0%	17.0%	0.1%	0.0%	15.4%	3.4%
	2009	180	**188**	1.5%	2.0%	0.0%	27.7%	6.3%	19.1%	0.1%	31.3%	1.4%	2.2%	4.1%	0.5%	0.0%	3.5%	0.1%
	2010	172	**133**	3.8%	5.1%	0.0%	3.5%	6.8%	0.0%	40.8%	27.4%	0.1%	1.8%	7.7%	0.5%	0.0%	2.3%	0.1%
	2011	178	**133**	23.0%	0.0%	0.0%	6.4%	15.3%	4.2%	0.9%	28.8%	1.5%	0.6%	8.9%	0.1%	0.0%	3.1%	7.1%
June																		
	1981	222	**18**	13.5%	0.0%	0.0%	1.7%	14.2%	0.0%	0.0%	47.1%	0.0%	15.0%	0.0%	0.0%	1.5%	6.9%	0.0%
	1982	197	**95**	7.5%	0.0%	2.2%	21.8%	3.4%	0.0%	13.1%	46.4%	0.0%	1.2%	2.5%	0.3%	0.0%	1.2%	0.5%
	1983	213	**21**	0.0%	0.0%	0.0%	0.0%	36.3%	0.0%	7.9%	32.8%	1.9%	14.0%	0.2%	0.4%	0.0%	6.4%	0.1%
	1984	182	**52**	4.6%	1.0%	1.6%	25.7%	20.9%	10.7%	3.8%	22.4%	2.9%	3.9%	0.7%	0.2%	0.0%	1.7%	0.0%
	1985	210	**69**	25.7%	4.9%	0.0%	0.8%	1.0%	0.0%	19.6%	35.0%	0.0%	4.9%	6.9%	0.4%	0.0%	0.8%	0.0%
	1998	209	**18**	0.0%	32.9%	0.0%	0.0%	2.0%	28.9%	2.9%	15.8%	0.0%	17.2%	0.3%	0.1%	0.0%	0.0%	0.0%
	1999	197	**134**	30.7%	0.0%	0.0%	4.0%	6.1%	0.4%	0.4%	34.9%	0.0%	16.4%	4.9%	0.5%	0.0%	1.7%	0.0%
	2000	195	**84**	0.0%	0.0%	0.7%	0.7%	0.5%	8.1%	9.4%	47.3%	0.0%	4.5%	25.3%	2.9%	0.0%	0.6%	0.0%
	2001	209	**40**	10.5%	0.0%	0.0%	0.0%	0.0%	4.1%	6.2%	59.6%	0.0%	7.9%	3.9%	0.7%	0.0%	7.0%	0.0%
	2002	196	**102**	24.4%	0.0%	0.4%	2.1%	4.6%	0.0%	14.6%	44.2%	0.0%	4.2%	3.4%	0.1%	0.0%	2.0%	0.0%
	2003	196	**107**	0.2%	0.0%	0.2%	0.2%	3.4%	13.0%	13.3%	57.1%	0.0%	2.0%	1.9%	0.3%	0.0%	8.4%	0.0%
	2004	200	**76**	0.0%	0.0%	0.3%	0.3%	0.4%	4.4%	34.2%	59.9%	0.0%	0.3%	0.1%	0.1%	0.0%	0.0%	0.0%
	2005*	180	**25**	0.0%	0.0%	0.0%	1.3%	0.0%	0.0%	48.0%	42.3%	0.0%	2.0%	4.7%	0.2%	0.0%	0.2%	1.4%
	2006	193	**87**	0.2%	0.0%	0.0%	0.5%	1.2%	3.8%	51.0%	30.5%	0.0%	6.1%	1.7%	0.2%	0.0%	4.7%	0.1%
	2007	190	**119**	0.2%	0.0%	0.0%	4.1%	3.4%	4.2%	35.2%	43.6%	0.2%	4.5%	1.6%	0.1%	0.0%	3.1%	0.0%
	2008	193	**262**	5.0%	0.0%	0.0%	10.9%	0.8%	2.3%	22.2%	43.8%	0.1%	2.1%	9.5%	0.0%	0.0%	3.3%	0.0%
	2009	199	**142**	0.0%	20.5%	0.0%	8.6%	4.7%	10.6%	18.5%	30.4%	0.1%	1.5%	1.0%	0.1%	0.0%	4.1%	0.0%
	2010	188	**112**	0.0%	5.0%	1.2%	0.4%	1.1%	6.2%	34.6%	42.8%	1.1%	0.9%	5.7%	0.0%	0.0%	1.0%	0.0%
	2011	199	**95**	0.0%	0.0%	0.0%	4.1%	11.1%	7.8%	22.1%	30.5%	1.8%	3.5%	10.6%	0.4%	0.0%	8.3%	0.0%

*includes 5 fish capture in last 3 days of May 2005
